# Development of a diagnostic DNA chip to screen for 30 autosomal recessive disorders in the Hutterite population

**DOI:** 10.1002/mgg3.206

**Published:** 2016-01-19

**Authors:** Barbara Triggs‐Raine, Tamara Dyck, Kym M. Boycott, A. Micheil Innes, Carole Ober, Jillian S. Parboosingh, Alexis Botkin, Cheryl R. Greenberg, Elizabeth L. Spriggs

**Affiliations:** ^1^ Departments of Biochemistry & Medical GeneticsUniversity of ManitobaWinnipegCanada; ^2^ Pediatrics & Child HealthUniversity of Manitoba745 Bannatyne Ave.WinnipegMB R3E 0J9Canada; ^3^ The Manitoba Institute of Child Health513‐715 McDermot Ave.WinnipegMB R3E 3P4Canada; ^4^ Clinical Biochemistry and GeneticsDiagnostic Services Manitoba at Health Sciences CentreWinnipegMB R3A 1R9Canada; ^5^ Children's Hospital of Eastern Ontario Research InstituteUniversity of OttawaOttawaON K1H 8L1Canada; ^6^ Department of Medical GeneticsAlberta Children's Hospital and Alberta Children's Hospital Research Institute for Child and Maternal HealthCumming School of MedicineUniversity of CalgaryCalgaryAlbertaCanada; ^7^ Departments of Human Genetics and Obstetrics and GynecologyThe University of ChicagoChicagoIllinois

**Keywords:** chip, Hutterite, diagnostic, mutation, APEX array, carrier screening

## Abstract

**Background:**

The Hutterites are a religious isolate living in colonies across the North American prairies. This population originated from approximately 90 founders, resulting in a number of genetic diseases that are overrepresented, underrepresented, or unique. The founder effect in this population increases the likelihood that Hutterite couples carry the same recessive mutations. We have designed a diagnostic chip on a fee‐for‐service basis with Asper Biotech to provide Hutterites with the option of comprehensive carrier screening.

**Methods:**

A total of 32 disease‐causing mutations in 30 genes were selected and primers were designed for array primer extension‐based testing. Selected mutations were limited to those leading to autosomal recessive disorders, maintaining its primary use as a test for determining carrier status.

**Results:**

The DNA chip was developed and validated using 59 DNA controls for all but one of the mutations, for which a synthetic control was used. All mutations were readily detected except for a duplication causing restrictive dermopathy where heterozygotes and homozygotes could only be distinguished by sequencing. Blinded testing of 12 additional samples from healthy Hutterites was performed by Asper Biotech using chip testing. All known mutations from previous molecular testing were detected on the chip. As well, additional mutations identified by the chip in these 12 samples were subsequently verified by a second method.

**Conclusions:**

Our analysis indicates that the chip is a sensitive and specific means of carrier testing in the Hutterite population and can serve as a model for other founder populations.

## Background

The North American Hutterite population is comprised of approximately 45,000 individuals living on colonies in the prairies of Canada and the United States (http://www.hutterites.org/). The Hutterites emerged during the Protestant Reformation in the 16th century as a religious isolate who believed in adult baptism (anabaptist), communal living including sharing of goods, and nonviolence (Hostetler 1974). Religious persecution led to their migration across central Europe and dramatic fluctuations in their population numbers. These population bottlenecks, coupled with reproductive isolation and population expansion, have resulted in a distinct group of genetic disorders that are overrepresented and in some cases unique to the Hutterites (Boycott et al. 2008).

Both demographic and genetic studies indicate that shortly after their founding, individuals from South Tyrol in Austria, Germany, and Switzerland settled to form a thriving Hutterite population in Moravia (Hostetler 1974). In 1874, continued religious persecution led 1265 Hutterites to emigrate to the United States (Eaton and Mayer 1953) where 443 Hutterites settled into three colonies, forming the basis of the Schmiedeleut, Lehrerleut, and Dariusleut divisions of Hutterite population structure. Those Hutterites who did not join the colonies integrated into the general population of the United States. During the First World War, Hutterites from the colonies moved to the Canadian Prairie provinces of Manitoba, Alberta, and Saskatchewan. Since that time, the Hutterites have been one of the most rapidly growing populations in the world, and have formed 107 colonies in Manitoba, 60 in Saskatchewan, 168 in Alberta, 2 in British Columbia, 54 in South Dakota, 7 in North Dakota, 9 in Minnesota, 50 in Montana, and 5 in Washington (http://www.hutterites.org/).

It is estimated that 89 individuals founded today's Hutterites (Nimgaonkar et al. 2000). The decreased genetic heterogeneity in the population, together with large family size and meticulous genealogical records, has greatly facilitated the identification of disease genes in this population through identity by descent mapping studies. As a result, the causative gene has been identified for at least 35 autosomal recessive disorders, and still others remain to be found (Boycott et al. 2008; Chong et al. 2012).

The same features that have facilitated the identification of disease genes have also resulted in an increased incidence of several genetic diseases, including some that are unique to the Hutterites. The carrier frequencies for a number of these disorders were recently examined in a large sample of Schmiedeleut Hutterites, and found to range from 1/6.5 to 1/48 in this group (Chong et al. 2012). Given the high carrier frequencies, the preexisting knowledge of the molecular basis of disease in this population, and strong endorsement from the Manitoba Hutterite ministers and constituents (personal communication, S. Maendel and Hutterite Brethren), we developed a screening tool that may be used by this population for family planning. With this in mind, herein, we describe the design and testing of a DNA chip that allows for the analysis of carrier status for 30 autosomal recessive conditions in the Hutterite population.

## Materials and Methods

### Ethical compliance

The University of Chicago Institutional Review Board provided approval to use the 12 DNA samples from the Hutterite population in an anonymized fashion.

### Disease identification for DNA chip

A list of genetic disorders described in the Hutterite population, and their molecular basis if known, was developed based on an exhaustive literature search and consultation with clinicians and researchers working on genetic disorders in this population. An expert panel made up of clinical geneticists, laboratory specialists, and basic scientists reviewed the list and selected autosomal recessive conditions that have clearly defined mutations and are clinically significant, for inclusion on the chip.

### DNA chip construction

The DNA chip was developed in consultation with Asper Biotech (http://www.asperbio.com/?s=Hutterite) on a fee‐for‐service basis. Oligonucleotides were designed for using the array primer extension (APEX) methodology which, in brief, utilizes Sanger sequencing that is limited to the addition of a single nucleotide to a primer on a microarray platform (Jaakson et al. 2003). The APEX method has been used extensively in a wide variety of clinical scenarios (Henderson et al. 2007). During the technical validation stage, control DNA samples containing mutations of the diseases of interest, were provided, without blinding, to verify the mutation detection. Mutations can be added to this chip for a fee, and the service for the analysis of samples using the chip is CLIA and ISO15189 certified.

### DNA samples

DNA samples from 59 controls known to be homozygous or heterozygous for a specific mutation were submitted to Asper Biotech for analysis, after all identifying information was removed. As the chip simultaneously detects 32 mutations, it was anticipated that mutations, other than the known ones, would be detected. In addition, 12 DNA samples from Schmiedeleut individuals, which had been analyzed as part of previous studies, were provided in a blinded fashion to assess the performance of the chip (Chong et al. 2012).

## Results

### Chip construction

An expert panel identified 30 autosomal recessive conditions in the Hutterite population to include for testing on a diagnostic chip (Table 1). The chip was constructed with oligonucleotides designed by Asper Biotech. To assess the specificity of the chip, 59 controls known to be heterozygous or homozygous for each mutation were sent for testing of the chip by Asper. As no known control was available for the *SLC5A5* c.1183G>A (p.G395R) mutation, which causes thyroid dyshormonogenesis I (also known as iodide transport defect), a synthetic DNA fragment was generated by Asper Biotech for specificity checks.

**Table 1:** Autosomal recessive disorders, and corresponding mutations, that have been included on the chip. Carrier frequencies were included if the mutation was either observed at least once in samples sent to Asper (Observed Freq.) and/or referenced in the literature (Literature Freq.)

Disorder	MIM	Phenotype	Gene	Mutation	Leut^a^	Observed Freq.^b^	Literature Freq.^d^	Ref.^f^
Arrhythmogenic cardiomyopathy	610476	Biventricular disorder causing heart failure, intractable arrhythmias with risk of sudden death in young adults	*DSC2*	c.1660C>T (p.Q554X)	D,L,S	1/8	1/10.6	(Gerull et al. 2013)
Bardet–Biedl syndrome	209900	Multisystem ciliopathy characterized by obesity, Retinitis pigmentosa, syndactyly polydactyly, hypogonadism, renal failure, and variable intellectual disability	*BBS2*	c.472‐2A>G	D,S	1/13	1/22–1/36	(Innes et al. 2010; Chong et al. 2012)
Beaulieu‐Boycott‐Innes syndrome	613680	Intellectual disability syndrome with microcephaly, characteristic facies and occasionally congenital heart or renal anomalies	*THOC6*	c.136G>A (p.G46R)	D	‐c	1/33–1/50	(Beaulieu et al. 2013; Boycott et al. 2010)
Bowen‐Conradi syndrome	211180	Severe IUGR, characteristic facies with prominent nose and micrognathia, contractures, profound developmental delay early lethality	*EMG1*	c.257A>G (p.D86G)	D,L,S	1/21	1/10	(Armistead et al. 2009; Lowry et al. 2003)
Carnitine palmitoyltransferase 1 deficiency	212500	Inborn error of fat metabolism causing hypoketotic hypoglycemia and “Reye‐like” syndrome with stressors such as fever and illness. Effective treatment if identified on newborn screening	*CPT1A*	c.2129 G>A (p.G710E)	D,S	1/16	1/15	(Prip‐Buus et al. 2001)
Cerebellar atrophy, short stature		Cerebellar hypoplasia, ataxia, short stature, global delay, and manganese deficiency	*SLC39A8*	c.112G>C (p.G38R)	D,S	1/21	Unknown^e^	(Boycott et al. 2015)
Combined pituitary hormone deficiency	262600	Deficiencies of growth hormone, thyroid hormone, gonadotropins, and occasionally +/− prolactin. Presents in infancy with FTT and growth failure.	*PROP1*	c.301_302delAG (p.L102 fs)	D,L,S	–	Unknown	(Boycott et al. 2008; Wu et al. 1998)
Congenital hyperinsulinism	256450	Presents usually with severe neonatal‐onset hyperinsulinemic hypoglycemia and seizures; may respond partially to diazoxide or may need pancreatic resection	*ABCC8*	c.823‐7T>A	S	–	Unknown	Stanley PC
Cranioectodermal dysplasia‐like		Scaphocephaly with or without sagittal craniosynostosis, ectodermal anomalies, short stature, developmental delay	*DPH1*	c.17T>A (p.M6K)	D	1/32	Unknown	(Loucks et al. 2015)
Cystic fibrosis	219700	Multisystem disorder with progressive lung disease and malaborption due to pancreatic insufficiency; newborn screening and early treatment recommended	*CFTR*	c.3302T >A (p.M1101K) c.1521_1523delCTT (p.I507_F508del)	D,L,S D,L,S	1/16 1/33	1/13.5 1/45.5	(Zielenski et al. 1993; Chong et al. 2012)
Dilated cardiomyopathy with ataxia syndrome	610198	Variable intellectual disability associated with gait and balance disturbance, short stature, genital anomalies in males, cardiomyopathy and arrhythmias often with 3‐methylglutaconic aciduria	*DNAJC19*	c.130‐1G>C	D,L,S	1/21	1/36	(Davey et al. 2006; Chong et al. 2012)
Hypophosphatasia	241500	Only one report in Hutterite baby, disorder of skeletal mineralization, with variable age of onset, caused by deficiency of alkaline phosphatase activity. Clinical trials with ERT show promising results	*ALPL*	c.1001G>A (p.G334D)	D	–	Unknown	(Gibson et al. 2000)
Joubert syndrome related disorder	614424	Hypotonia, global developmental delay/intellectual disability, renal anomalies, variable encephalocele, and polydactyly, molar tooth sign on MRI	*TMEM237*	c.52C>T (p.R18X)	D,S	1/16	1/12.5	(Huang et al. 2011; Chong et al. 2012; Boycott et al. 2007)
Leigh disease	256000	Severe disorder of energy metabolism with encephalopathy and loss of motor and cognitive skills	*NDUFS4*	c.393dupA (p.K131 fs)	D,S	1/13	Unknown	(Boycott et al. 2008)
Limb girdle muscular dystrophy 2H	254110	Slowly progressive late‐onset muscular dystrophy with mainly proximal weakness; not associated with cardiomyopathy	*TRIM32*	c.1459G>A (p.D487N)	D,S	1/31	1/6.5	(Frosk et al. 2002; Chong et al. 2012)
Limb girdle muscular dystrophy 2I	607155	Proximal myopathy similar to LGMD2H but earlier age of onset, variable rate of progression, and cardiomyopathy which can be presenting manifestation	*FKRP*	c.826C>A (p.L276I)	D,S	1/7	1/9.5	(Frosk et al. 2005; Chong et al. 2012)
Limb girdle muscular dystrophy 2S	615356	Muscular dystrophy with variable movement disorder and abnormal posturing of limbs; may have intellectual disability	*TRAPPC11*	c.1287 + 5G>A	D,S	1/8	1/14	(Bogershausen et al. 2013)
Methylmalonic aciduria	251000	Severe inborn error with neonatal presentation of metabolic acidosis and hyperammonemia; isolated MMA 20 mut0 defect of methylmalonyl‐CoA mutase unresponsive to B12 and often fatal	*MUT*	c.1420C>T (p.R474X)	D	1/63	Unknown	(Worgan et al. 2006)
Nephronophthisis‐juvenile	256100	Cystic renal disease‐causing renal failure in childhood or adolescence	*NPHP1*	290 Kb deletion c.1918delA (p.R640fs)	D	–	Unknown	(Hildebrandt et al. 1997; Boycott et al. 2008)
Oculocutaneous albinism type 1A	203100	Disorder of melanin biosynthesis with generalized reduction in pigmentation of hair, skin and eyes, fovea hypoplasia and optic nerve defect; severely reduced vision	*TYR*	c.272G>A (p.C91Y)	D,L,S	1/9	1/7	(Chong et al. 2012; Boycott et al. 2008)
Sensorineural deafness	220290	Isolated congenital sensorineural deafness	*GJB2*	c.35delG (p.G12fs)	D,S	1/65	1/28	(Chong et al. 2012)
Nonsyndromic mental retardation	614020	Intellectual disability with no associated distinguishing clinical features or metabolic abnormality known	*TECR*	c.545C>T (p.P182L)	S	1/63	1/14.5	(Caliskan et al. 2011; Chong et al. 2012)
Restrictive dermopathy	275210	Lethal disorder presenting at birth with characteristic very tense, translucent skin, severe arthrogryposis and lung hypoplasia	*ZMPSTE24*	c.1085dupT (p.L362fs)	D,S	1/21	1/15.5	(Loucks et al. 2012; Chong et al. 2012)
Segawa syndrome	605407	Inborn error of metabolism presents as progressive movement disorder with variable dystonia, tremors and abnormal posturing; low CSF neurotransmitters, good response to levodopa; normal intelligence	*TH*	c.1481C>T (p.T494M)	D	–	Unknown	(Boycott et al. 2008)
Sitosterolemia	210250	Also known as “phytosterolemia”, caused by excessive absorption and decreased excretion of plant sterols leading to premature atherosclerosis; responds to ezetimide	*ABCG8*	c.320C>G (p.S107X)	S	1/16	1/12	(Wang et al. 2004; Chong et al. 2012)
Succinylcholine sensitivity	177400	“pseudocholinesterase” deficiency leads to delayed metabolism of the muscle relaxant drug succinylcholine used in general anesthesia; causes prolonged apnea post operatively	*BCHE*	c.293A>G (p.D98G)	S	1/21	Unknown	(Zelinski et al. 2007)
Thyroid dyshormonogenesis I	274400	Congenital hypothyroidism identified on newborn screening by low T4; iodine transport defect; excellent response to treatment	*SLC5A5*	c.1183G>A (p.G395R)	S		Unknown	(Kosugi et al. 1999)
Usher syndrome Type 1B	276900	1 of 2 forms of Usher syndrome seen in Hutterites with congenital sensorineural deafness and progressive visual impairment due to retinitis pigmentosa	*MYO7A*	c.52C>T (p.Q18X)	D	–	Unknown	(Zhou et al. 2012)
Usher syndrome Type 1F	602083	Similar to Usher syndrome 1B	*PCDH15*	c.1103delT (p.L368 fs)	S	1/21	1/40	(Chong et al. 2012; Alagramam et al. 2001)
VLDLR with cerebellar hypoplasia	224050	Early‐onset ataxia and intellectual disability, simplified gyral pattern and distinctive cerebellar hypoplasia	*VLDLR*	Deletion of *VLDLR* gene	D,L,S	–	1/15	(Boycott et al. 2005; Glass et al. 2005)

a D‐Dariusleut; L‐Leherleut; S‐Schmeideleut refer to the leuts in which the mutation has been detected.

b Carrier frequency observed in samples tested on the chip. Mutations were excluded from the calculation of the carrier frequency if the sample was ascertained as a positive control for that disease.

c Indicates the mutation was not detected in any of our samples.

d Literature frequency refers to frequencies that have been determined by testing or estimated based on disease incidence and reported in the literature.

e Unknown indicates that no estimate of the frequency has been made in the literature to our knowledge.

f References to the frequencies in the previous column or the first report of the mutation.

**Figure 1:**
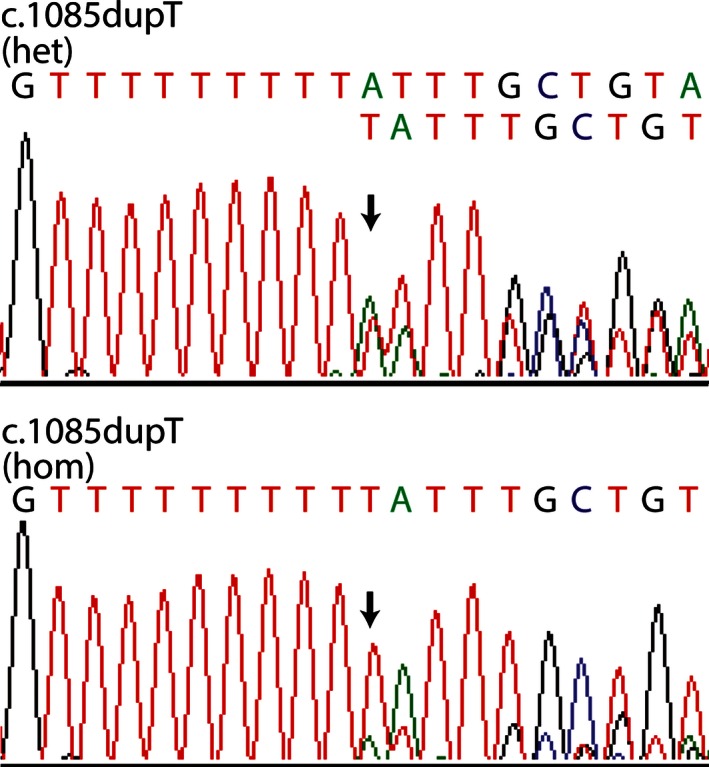
Sequence of the c.1085dupT mutation in *ZMPSTE24*. Sequencing of the region differentiated the heterozygous (Het) and homozygous (Hom) samples for the c.1085dupT.

All but one mutation were reliably detected as either wild type, heterozygous, or homozygous. This mutation, *ZMPSTE24* c.1085dupT causing restrictive dermopathy, a lethal disorder, could only be detected as either absent or present with the chip. This is not clinically relevant because if the chip test is positive for restrictive dermopathy, this would indicate heterozygosity of the adult being tested, as individuals with restrictive dermopathy who are homozygous for the *ZMPSTE24* c.1085dupT mutation die early in life (Loucks et al. 2012). Nonetheless, further analysis by DNA sequencing would allow a heterozygous and a homozygous genotype to be differentiated in situations where a clinical diagnosis needs to be clarified (Fig. 1).

In addition to the mutations that were expected in the 59 control samples, on average, 1.2 additional mutations were identified. Three samples were homozygous for diseases other than that for which they served as a positive control, including two affected with Joubert syndrome‐related disorder and one with limb girdle muscular dystrophy type 2I. More than one disease diagnosis may have already been documented as present in these individuals, but because the samples were de‐identified, this was not possible to follow‐up.

To test the sensitivity of the chip, 12 Schmiedeleut Hutterite samples, that had previously been sequenced or tested for a number of mutations as part of previous studies, were submitted in a blinded fashion to Asper for testing (Chong et al. 2012). In total, 10 different mutations were identified in these samples. All but one mutation, where a lack of DNA prevented its validation, was confirmed to be present by comparison to previous whole genome sequencing, previous genotyping results, or through a secondary method. No mutations that had previously been identified by another method were missed by the chip. Overall, there was a 100% concordance of the chip results with the results of other methods.

When mutations additional to those expected in the 59 control samples were combined with those identified in the 12 additional samples used to test the sensitivity of the chip, it became clear that the carrier frequencies of the different mutations were variable in our samples (Table 1). We decided to compare the results to those of previous studies where a carrier frequency had been estimated (Table 1) in order to identify any obvious discrepancies, and to provide new information where none was previously available. One obvious difference was the absence of the *VLDLR* gene deletion in our samples when it had previously been estimated to have a carrier frequency of 1/15 in the Alberta Hutterites (Dariusleut and Lehrerleut) (Glass et al. 2005). The absence of the *VLDLR* deletion was therefore confirmed in the 12 anonymous samples by a second method, and this discordance may reflect a lower carrier rate in the Schmiedeleut Hutterite population.

## Discussion

We have developed a reliable tool for carrier screening of 30 autosomal recessive conditions in the Hutterite population. Further, additional mutations can be added to this chip as new mutations are identified. This test is currently available through Asper Biotech and is quite affordable at $235 EUR (~$250 USD). Given that the price of testing a single known mutation is similar at $250 USD, the use of the chip to interrogate 32 mutations simultaneously is highly cost‐effective. We anticipate that this test will primarily be used on a voluntary basis for adult individuals who are considering marriage or for couples planning a family. It might also be used in unique situations where the diagnosis of a previously affected child is unclear or if multiple diseases on the chip are part of a differential diagnosis for an affected individual.

This chip, although broad in scope, does not currently provide comprehensive carrier screening for the Hutterite population. One of the clinically significant autosomal recessive diseases with a high carrier frequency that was not included is spinal muscular atrophy (SMA). A founder haplotype for SMA in Hutterites has been described that could be identified using 26 single‐nucleotide polymorphisms (Chong et al. 2011). Unfortunately, our resources at the time of development did not allow the incorporation of this test onto the chip. Also, the molecular basis of Maple Syrup Urine Disease, BCKDHB gene, c.595_596delAG (Mroch et al. 2014), and a second Joubert‐like condition resulting from mutations in *CSSP1*(Shaheen et al. 2014) were recently reported and could now be added. No doubt additional mutations will continue to be identified that could make this an even more comprehensive test.

Among the disorders tested on this chip, several are relatively unique to the Hutterite population. For example, Bowen–Conradi syndrome, limb girdle muscular dystrophy type 2H, dilated cardiomyopathy with ataxia, Beaulieu‐Boycott‐Innes syndrome, and nonsyndromic mental retardation due to *TERC1* mutations have not, or rarely been molecularly confirmed in non‐Hutterites. In the case of Bowen–Conradi syndrome, no mutation in *EMG1* has been found in non‐Hutterite infants, who were thought to have the condition based on clinical presentation (Triggs‐Raine et al., unpubl.data). Further, many of the mutations in Table 1 are unique to the Hutterite population, reflecting their unique founder ancestry.

The Hutterites, together with the Mennonites and Amish, arose during the Protestant Reformation in central Europe. These groups were derived during the 16th and 17th centuries from similar ancestral populations, and thus it is not surprising that some mutations are shared between these populations. A summary of the disorders found in the Anabaptist groups is at http://www.biochemgenetics.ca/plainpeople/(Payne et al. 2011). As an example, the c.1085dupT mutation in *ZMPSTE24* that was reported in Old Colony Mennonites is also recently reported in Hutterites (Loucks et al. 2012). Similarly, the c.1001G>A mutation in *ALPL* was originally found to cause hypophosphatasia in Mennonites (Greenberg et al. 1993) and subsequently detected in at least one Hutterite infant.

Many of the mutations on the CHIP were identified in one or more of the positive controls and/or anonymous samples that we tested (Table 1). One unexpected finding was a high carrier frequency (1/13) for the c.393dupA mutation in *NDUFS4* causing Leigh syndrome. Given this finding, it would be important for physicians who work with the Hutterite population to consider this disease on the differential diagnosis in infants presenting with progressive neurodegeneration. The carrier frequency for succinylcholine sensitivity was also unknown, and this study suggests a frequency of approximately 1/21 Hutterites. As a result of this relatively high carrier frequency, the use of succinylcholine in induction of general anesthesia is avoided in children of Hutterite descent at the Health Sciences Centre in Manitoba. The majority of the control and anonymized samples were from the Schmiedeleut and Dariusleut groups, therefore resulting in a bias toward detecting the mutations in those leut (Table 1). In fact, 10 mutations were not identified in either our controls or anonymous samples, suggesting that these mutations may have a low carrier rate, or that they are private to the Lehrerleut.

A comprehensive study of the changes in the frequency of autosomal recessive disease‐causing mutations in the Schmiedeleut population demonstrated an increase in the frequency of some disease‐causing mutations (Chong et al. 2012). The release of the results of this study indicated that Hutterite individuals want carrier information (Anderson et al. 2014), consistent with the interest expressed by Schmiedeleut Hutterites in Manitoba. Although future research is needed to assess the impact of comprehensive carrier testing in the population, it is clear that this approach has the potential to provide information that will empower this group.

## Conflict of Interest

None declared.
